# Nattokinase crude extract enhances oral mucositis healing

**DOI:** 10.1186/s12903-021-01914-4

**Published:** 2021-10-30

**Authors:** Junyao Zhang, Yu Tang, Tao Yuan, Mengting Yang, Wenjing Fang, Li Li, Fei Fei, Aihua Gong

**Affiliations:** Department of Cell Biology, School of Medicine, Jiangsu University, 301 Xuefu Road, Zhenjiang, 212003 China

**Keywords:** Nattokinase, *Bacillus subtilis*, Mucosal epithelium, Oral mucositis healing

## Abstract

**Background:**

Nattokinase (NK) is a promising alternative in the prevention and treatment of cardiovascular diseases due to its potent fibrinolytic activity. In this study, we investigated the effect of crude nattokinase extract on the healing of acetic acid-induced oral mucositis in mice.

**Methods:**

*Bacillus subtilis* culture media (BSCM) was isolated into the supernatant, named nattokinase crude extract (NCE), and the pellet was named *Bacillus subtilis* mass (BSM). An oral mucositis model was established in mice by applying 50% glacial acetic acid to the buccal mucosa. According to the treatment conditions, the mice were divided into BSCM, NCE, BSM and phosphate buffered saline (PBS) groups. The weight of the mice, oral mucositis healing score and histopathological examination were used to evaluate the treatment.

**Results:**

Fibrinolytic activities of BSCM, NCE and BSM were approximately 8069, 10,800 and 80 U/ml, respectively. The weight gain of mice in the NCE group was significantly different from the PBS group after three days’ treatment (*p* < 0.05). The oral mucositis score of NCE group was significantly higher than other groups (*p* < 0.05). The differences in histopathology scores between the NCE and other groups were statistically significant (*p* < 0.01).

**Conclusions:**

NCE could possess remarkable potential to reduce pain and promote oral mucositis healing with minimal safety concerns. In this study, we first report that NCE from the supernatant of *Bacillus subtilis* can promote the healing of oral mucositis, which extends the application scope of NK.

## Background

Oral mucositis is one of the adverse effects associated with upper body radiotherapy or chemoradiotherapy [1, 2]. During the initiation phase of mucositis, patients begin to develop erythema followed by focal areas of oral mucosal desquamation [3]. Then the progress of mucositis is prolonged and severe, the integrity of the mucosa is destroyed, and lesions begin to form [4]. These lesions can cause mild to severe pain, which often leads to swallowing disorders, result in weight loss in mice [5–7]. The pain is mainly stimulated by inflammatory mediators such as prostaglandins, 5-hydroxytryptamine and bradykinin secreted by inflammatory cells [8]. At present, a combination of treatments, such as local rinses with 2% viscous lidocaine solution, mouthwash preparations and other systemic analgesics are used to control pain [9]. However, most commercially available mouthwashes contain alcohol and other ingredients, which sensitize the lesion and make patients uncomfortable. Therefore, there is a dire need to develop mild strategies to promote oral mucositis healing.

In 1987, Sumi et al. [10] first named a novel fibrinolytic enzyme NK, which was found in natto, a typical and popular fermented soybean food in a traditional Japanese diet. NK is the most important extracellular enzyme produced by *Bacillus subtilis natto* [11]. Both in vitro and in vivo studies have confirmed that NK has potent fibrinolytic activity [12]. Thus, NK is now considered an efficient, secure and economic enzyme that draws central attention in thrombolytic drug studies [13–16]. In addition, NK is also used in the treatment of Alzheimer’s disease, vitreoretinal disorders and some tumours [17, 18]. Clinical trials have also proven that increasing the intake of nattokinase is effective in preventing and treating hypertension [19].

Recent study revealed that NK exerts its anti-inflammatory activity by inhibiting LPS-induced activation of TLR4 and NOX2, which may help patients relieve the pain [20]. According to our previous research, NK enhances cell stemness and induces epithelial cell regeneration and angiogenesis, thereby promoting wound healing [21]. These results indicate that NK might be effective in oral mucositis healing.

In this study, we aimed to investigate the changes in body weight, inflammation and the progression of epithelial regeneration in mice to determine the role of NCE in the treatment of oral mucositis.

## Methods

### Ethical approval

All procedures performed in this study were in accordance with the relevant guidelines and regulations. The animal study was reviewed and approved by the Medical Ethics Committee and Ethics Committee for Experimental Animals of Jiangsu University (IRB protocol number: 2012258). Additionally, the authors followed all guidelines of The Animal Research: Reporting in vivo Experiments guidelines (ARRIVE).

### Medium and culture conditions

The production of nattokinase mainly involves the following two steps: strain activation and fermentation. First, *Bacillus subtilis natto* was inoculated in seed culture medium (sodium chloride 2%, peptone 1%, yeast extract 0.5% and 100 mL of deionized water). The seed cultures were inoculated (0.5%, v/v) into a fermentation medium for NK production after cultivation in a thermostat at 37 °C for 24 h. The fermentation medium comprised 5% red bean powder, 2% glucose, 1% sodium chloride, 0.1% dipotassium hydrogen phosphate, 0.1% potassium dihydrogen phosphate, 0.01% calcium chloride and 0.05% magnesium sulphate. The fermentation media was contained in a conical flask and incubated in a shaker at 225 rpm at 37 °C for 72 h.

### Isolation of NCE

NCE was extracted from the fermentation medium, or BSCM. BSCM was centrifuged for 8 min at 12,000 × g, the liquid supernatant was named NCE, and the pellet was resuspended in PBS and named BSM.

### Preparation of agarose-fibrin plate

The fibrinolytic activity of NCE and other components was measured by the agarose-fibrin plate method. Briefly, the fibrin plate was made with a fibrinogen and agarose solution. Urokinase was used as the standard. The samples were dropped in the hole on the plate. The plate was incubated at 37 °C for 18 h. Finally, the fibrinolytic activity was determined from a standard curve.

### Mice oral mucositis model and treatment

Adult male ICR mice (8 weeks old; weighing 25 ± 2 g) were purchased from the Animal Centre of Jiangsu University (Zhenjiang, China), housed in individual plastic cages and subjected to a 12-h light–dark cycle with 60% relative humidity at 25 ± 2 °C. A diameter of 2-mm piece of filter paper was soaked in 50% acetic acid and placed in the labial fornix region of the inferior incisors of anaesthetized mice for 60 s to form the lesion [22, 23]. The mice were grouped into four groups, five mice one group. PBS treatment was used as a control group, and the other groups were treated with BSCM, NCE and BSM respectively. At 3, 6 and 10 days after treatment, we photographed oral mucositis lesions. Three calibrated and blinded observers were involved to evaluated the oral mucositis healing through specific evaluation criteria, and the interobserver variability was calculated. (0: model initial lesion, redness and grey pseudomembrane; 1: lesion destruction, unbleached pseudomembrane, obvious exudate, and redness; 2: lesion convergence, slight redness and less exudate; 3: significantly smaller lesion, a little exudate, a little redness; 4: completely healed lesion). Finally, the mice were sacrificed and the lesion tissue were resected for further analysis.

### Measurement of mice weight

We recorded the weight of mice daily after treatment. The relative weight = [(*dn *− *d0*)/*d0*] (*d0* is original weight of mice, and *dn* is the weight of mice were recorded after n days).

### Histopathological examination

The lesion tissue was fixed with 4% paraformaldehyde (pH 7.4), gradually dehydrated and embedded in paraffin. Four-micrometer-thick sections were obtained with a microtome (Leica, RM2145, Germany) and stained with haematoxylin and eosin(H&E) for assessing inflammatory response and histological changes. Images were captured with a microscope (Zeiss, Axio Scope A1, Germany). Three calibrated and blinded observers were involved to evaluated the histopathological images and the interobserver variability was calculated. H&E stained lesions were analysed and scored through following evaluation criteria (0: vasodilatation, leukocyte infiltration and oral epithelial destruction; 0.5: vascular contraction, leukocyte decline and oral epithelial destruction; 1: vasoconstriction, leukocyte decline, oral epithelial destruction and a small amount of lymphocytes; 1.5: vascular obviously destroyed, leukocyte not seen, oral epithelial destruction and a large number of lymphocytes; 2: normal vascular, leukocyte are not seen and a small number of lymphocytes; 2.5: normal vascular, leukocyte are not seen, healed oral epithelium and lymphocytes are not seen.).

### Statistical analysis

Data are shown as the mean ± SD. Comparisons between groups were analysed using Student’s t test (two groups) or one-way analysis of variance (ANOVA; multiple groups) using Prism 5.0 software (GraphPad, San Diego, CA). P values < 0.05 were considered significant.

## Results

### Detection of fibrinolytic activity

**Fig. 1:**
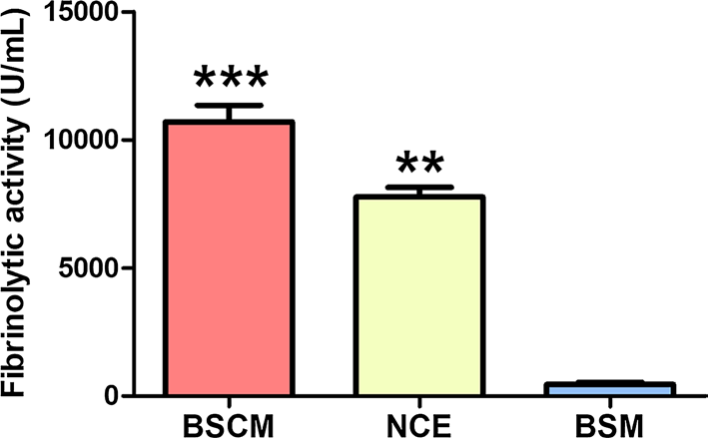
Fibrinolytic activity detection with agarose-fibrin plates. (n = 3, **p < 0.01, ***p < 0.001)

The fibrinolytic activity of different components from *Bacillus subtilis* culture media was detected with agarose-fibrin plates. The results showed that the fibrinolytic activity of NCE was approximately 8069 U/ml, the fibrinolytic activity of BSCM was approximately 10,800 U/ml, and the fibrinolytic activity of BSM was only 80 U/ml (Fig. 1).

### NCE increases the relative weight of the oral mucositis mice

**Fig. 2:**
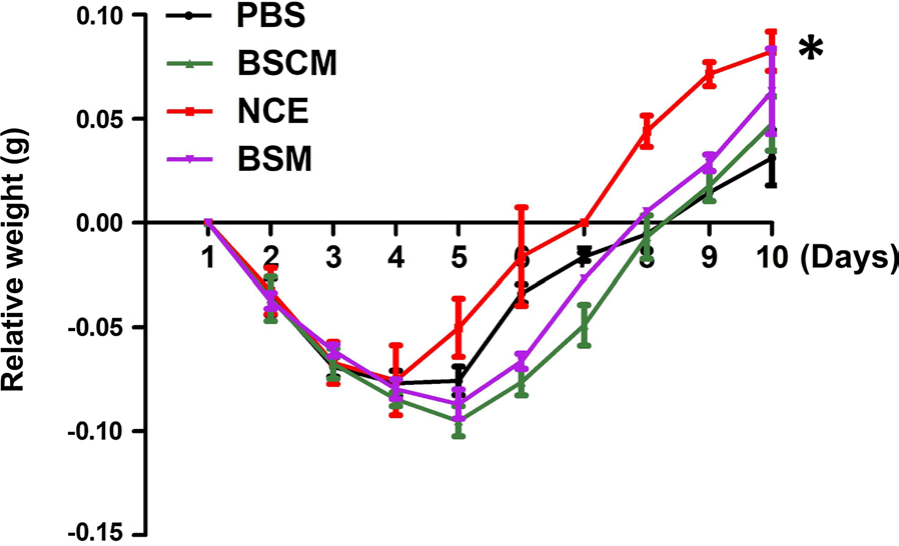
NCE promotes the weight gain of the model mice. (NCE group compared with PBS group, *p < 0.05)

An oral mucositis model was constructed in mice, and the relative weight of the mice after administration was used to evaluate the degree of oral mucositis healing. As shown in Fig. 2, the weight gain of mice in the NCE group was significantly different from the PBS group after three days’ treatment (*p* < 0.05) The body weight of mice in other groups did not increase until the 5th day after treatment.

### NCE accelerates oral mucositis healing

**Fig. 3:**
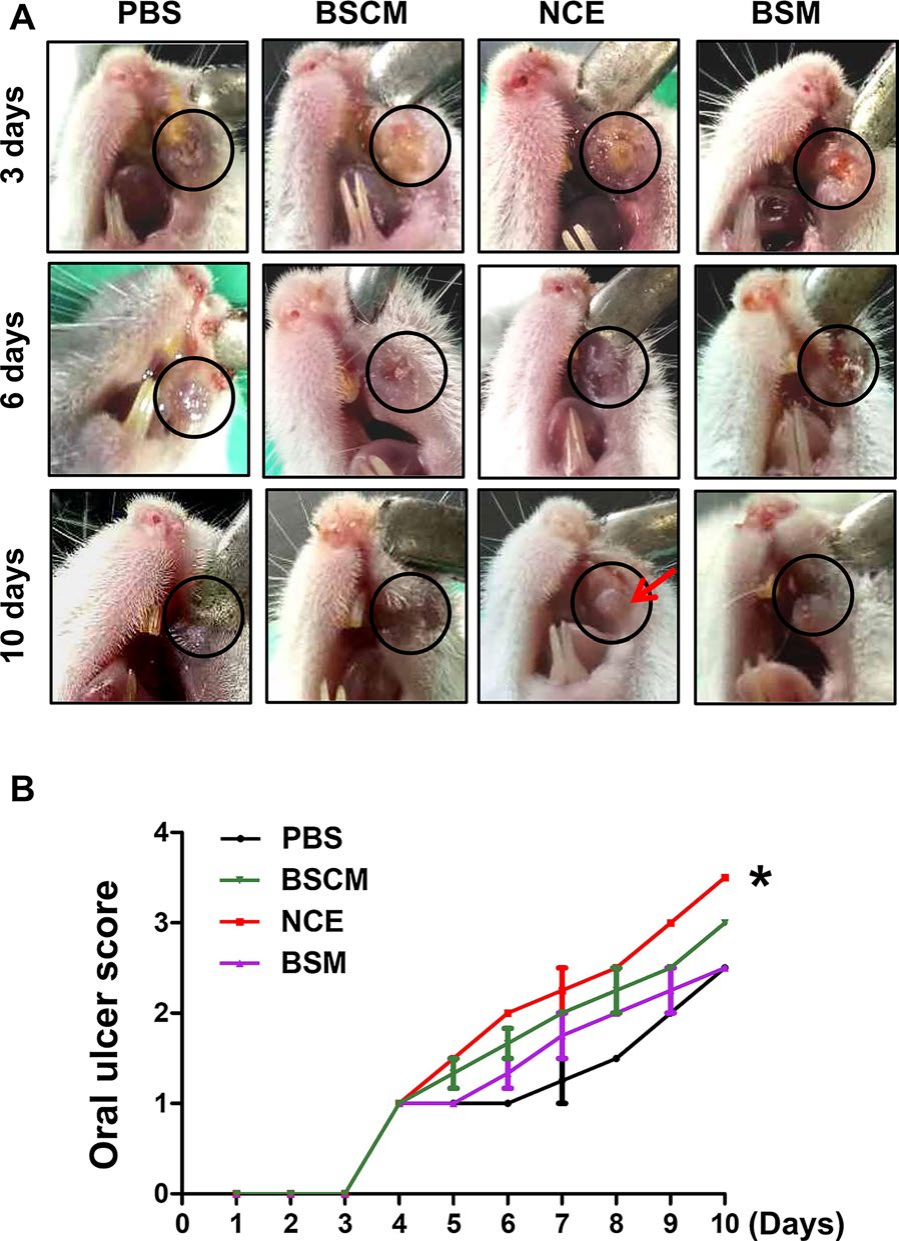
NCE promotes oral mucositis healing. **a** Representative images of oral mucositis lesion on the 3rd, 6th and 10th days after treatment with PBS, BSCM, NCE and BSM. The circles mark the location of the lesion and the arrow indicates the healing of oral mucositis. **b** Oral mucositis scored through evaluation criteria (*p < 0.05)

The general pictures showed that the area of oral mucositis in the NCE group was reduced and the exudate was less than that in the other three groups on the 3rd day after treatment. Surprisingly, the oral mucositis lesion in the NCE group healed compared with the other three groups on the 6th day after treatment. PBS did not promote oral mucositis healing (Fig. 3a). The score of oral mucositis was analysed as presented in Fig. 3b, the score of NCE group was significantly higher than the PBS group (*p* < 0.05). The comparison of oral mucositis scores is displayed in Table 1.

**Table 1:** Comparison of oral mucositis scores between NCE group and PBS group

Group	Time									
	Day 1	Day 2	Day 3	Day 4	Day 5	Day 6	Day 7	Day 8	Day 9	Day 10
PBS (N = 3)	–	–	–	1	1	1	1.25	1.5	2	2.5
NCE (N = 3)	–	–	–	1	1.5	2	2.25	2.5	3	3.5
BSM (N = 3)	–	–	–	1	1	1.3	1.75	2	2.25	2.5
BSCM (N = 3)	–	–	–	1	1.3	1.7	2	2.25	2.5	3
*P* value^a^						< 0.05				< 0.05

^a^Comparison between NCE and PBS

### NCE enhances mucosal epithelial regeneration in oral mucositis

**Fig. 4:**
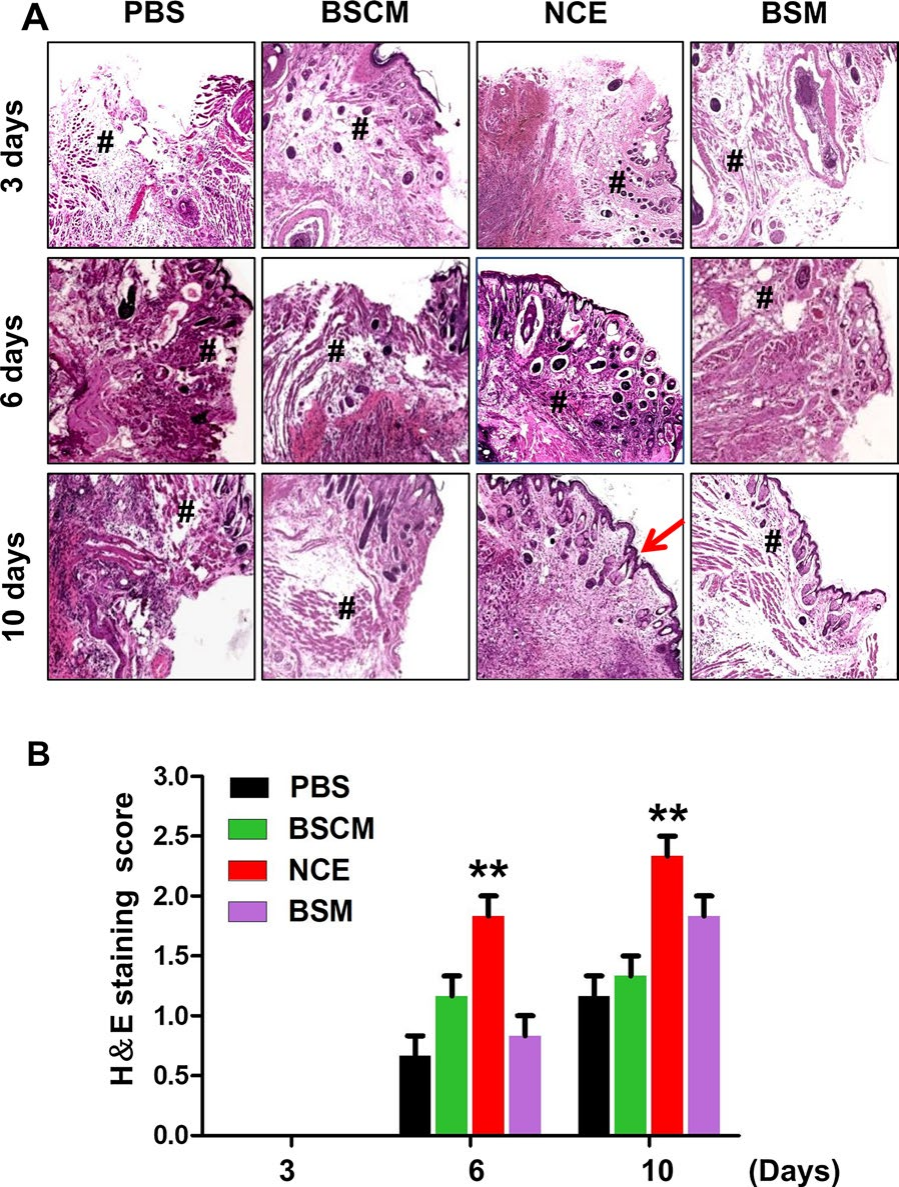
NCE enhances re-epithelialization of oral mucositis. **a** Representative photomicrograph of H&E staining of lesion tissue on the 3rd, 6th and 10th days after treatment. The arrow indicates re-epithelialization, **#** indicates inflammation. **b** Histopathological score of each group (**p < 0.01)

The results of H&E staining showed that the epithelial layer of each group was destroyed at 72 h after acetic acid treatment, accompanied by severe inflammatory cell infiltration. After 3 days of treatment, the NCE group had smaller lesions and reduced inflammatory cell infiltration compared with the other three groups. On the 6th day after the treatment, the epithelium in the NCE group recovered completely without inflammatory cell infiltration, while in the other three groups, the epithelium was not repaired, and there was still inflammatory cell infiltration (Fig. 4a). As depicted in Fig. 4b, the difference in histopathology score between the NCE group and the PBS group was statistically significant (*p* < 0.01).

## Discussion

Here, we confirmed the role of NCE in oral mucositis healing. We found that NCE was closely correlated with body weight changes, inflammation control and epithelial regeneration in mouse oral mucositis model.

Clinical features of oral mucositis include diffuse mucosal erythema and ulcerations, accompanying with mouth and throat pain [24], which interfere with eating and swallowing [25]. In particular, eating cold, hot, acidic and irritant foods often causes severe pain, which seriously affects quality of life [26]. Many interventions have been developed to treat oral mucositis, such as oral zinc sulphate, chlorhexidine, honey and laser therapy [27–29]. However, these interventions will bring additional pain to the patients. In this study, we found that the mouse weight of the NCE-treated group was increased significantly on the 4^th^ day after administration compared with that of the other groups, which indicated that NCE might relieve pain caused by oral mucositis and increase the mouse weight associated with eating and drinking.

Previous studies have confirmed that excessive inflammatory cells and factors participate in the formation of oral mucositis and stimulate sensory nerve endings [30]. Anti-inflammatory agents are regarded as the first-line agents to control the symptoms of oral mucositis. In our study, according to histological findings, the NCE group had better epithelial healing and moderate chronic inflammation on the 6th day than the other groups, which indicated that NCE can reduce excessive inflammatory responses and has a therapeutic effect on oral mucositis.

Speeding up the healing process, reducing pain and the chance of recurrence are the main aims of current therapy [31]. Several natural compounds can be administered as dietary supplements for the treatment of dermal and oral mucositis, our research extend this list [32]. As a food, NK has been widely proven to be safe, and toxicological assessments have also shown that the oral consumption of NK is of low toxicological concern [16].

Oral mucositis healing is a multifactorial process that involves inflammation, cell proliferation, migration and connective tissue remodelling [6]. Although we have demonstrated that NCE is effective in treating oral mucositis, the exact molecular mechanisms involved are still unknown and deserve further study.

## Conclusions

NCE could possess remarkable potential to reduce pain and promote oral mucositis healing with minimal safety concerns. In this study, we first report that NCE from the supernatant of *Bacillus subtilis* can promote the healing of oral mucositis and should be a valid alternative to the conventional treatment.

## Data Availability

The various raw data and methods used to support the findings of this study are available from the corresponding author upon request.

## Abbreviations

BSCM: *Bacillus subtilis* culture media
BSM: *Bacillus subtilis* mass
H&E: Haematoxylin and eosin
NCE: Nattokinase crude extract
NK: Nattokinase
PBS: Phosphate buffer saline
